# Association between mode of delivery and body mass index at 4-5 years in White British and Pakistani children: the Born in Bradford birth cohort

**DOI:** 10.1186/s12889-021-11009-y

**Published:** 2021-05-26

**Authors:** Eleanor Ralphs, Lucy Pembrey, Jane West, Gillian Santorelli

**Affiliations:** 1 London School of Hygiene and Tropical Medicine, Keppel Street, London, UK; 2 Bradford Institute for Health Research, Bradford Teaching Hospitals Foundation Trust, Bradford, UK

## Abstract

**Background:**

Globally, it is becoming more common for pregnant women to deliver by caesarean section (CS). In 2020, 31% of births in England were CS, surpassing the recommended prevalence of CS. Concerns have been raised regarding potential unknown consequences of this mode of delivery.

Childhood adiposity is also an increasing concern. Previous research provides inconsistent conclusions on the association between CS and childhood adiposity. More studies are needed to investigate the consequences of CS in different populations and ethnicities. Therefore, this study investigates the association between mode of delivery and BMI, in children of 4–5 years and if this differs between White British (WB) and Pakistani ethnicities, in Bradford UK.

**Methods:**

Data were obtained from the Born in Bradford (BiB) cohort, which recruited pregnant women at the Bradford Royal Infirmary, between 2007 and 2010. For these analyses, a sub-sample (*n* = 6410) of the BiB cohort (*n* = 13,858) was used.

Linear regression models determined the association between mode of delivery (vaginal or CS) and BMI z-scores at 4–5 years. Children were categorised as underweight/healthy weight, overweight and obese, and logistic regression models determined the odds of adiposity. Effect modification by ethnicity was also explored.

**Results:**

Multivariable analysis found no evidence for a difference in BMI z-score between children of CS and vaginal delivery (0.005 kg/m^2^, 95% CI = − 0.062–0.072, *p* = 0.88). Neither was there evidence of CS affecting the odds of being overweight (OR = 1.05, 95% CI = 0.86–1.28, *p* = 0.65), or obese (OR = 0.98, 95% CI = 0.74–1.29, *p* = 0.87). There was no evidence that ethnicity was an effect modifier of these associations (*p* = 0.97).

**Conclusion:**

Having CS, compared to a vaginal delivery, was not associated with greater adiposity in children of 4–5 years in this population. Concerns over CS increasing adiposity in children are not supported by the findings reported here using the BiB study population, of both WB and Pakistani families.

**Supplementary Information:**

The online version contains supplementary material available at 10.1186/s12889-021-11009-y.

## Background

Delivery by caesarean section (CS) is increasing globally. Using data from 150 countries from 1990 to 2014, longitudinal analysis suggests that CS represent 18.6% of all births [1]. In England, CS rates rose from 23 to 31%, between 2004 and 2020 [2].

Research suggests CS rates are increasing due to protective effects against fetal death [3] and to avoid adverse impacts of macrosomia in obese and diabetic mothers [4]. Also CS is sometimes perceived as more convenient, less painful and more profitable for private hospitals [4]. Contributions to such CS rates additionally arise from the cohort of women who have had one previous CS [5].

However, the rise in CS has aroused alarm due to the lack of knowledge on the short- and long-term risks. The World Health Organisation recommends CS should ideally only be undertaken when medically necessary and that CS rates higher than 10% are not associated with reductions in maternal and newborn mortality rates [6]. Some evidence suggests those who have undergone CS have just over twice the odds of severe maternal morbidity, compared to those experiencing vaginal deliveries [3]. CS has also been associated with other complications, such as a higher risk of immune and metabolic disorders in children [7], and offspring overweight and obesity [8–11]. The latter complication will be investigated in this report.

Overweight and obesity in England was prevalent in 22.6% of children aged 4–5 years, in 2018–2019; more specifically 23.1% in White British (WB) children and 19.9% in Pakistani children [12].

In the first 6 months of life, the colonisation and diversity of gut microbiota is associated with the mode of delivery [13]. Those born vaginally have a higher abundance of Bifidobacteria and Bacteroides than those born by CS [13]. These bacteria genera have a protective effect against being overweight as they are well equipped to obtain nutrients from breast (or formula) milk oligosaccharides [14]. Additionally, there is evidence to suggest the gut microbiota of a child born by CS is more abundant in *Staphylococcus aureus*, which has been associated with the development of obesity [14, 15]. It is important to note that guidelines endorse the use of prophylactic antibiotics for women undergoing CS, to prevent wound infection [16].

However, in children over the age of 6 months, there is very weak evidence of an association between mode of delivery and gut microbiota [13, 17], suggesting that the protective effect of vaginal deliveries against adiposity attenuates through early childhood. This conflicts with some evidence of an association between mode of delivery and BMI found in adult life [18], highlighting additional mechanisms might explain this association.

Previous research presents mixed results. One systematic review concluded children delivered by CS had higher odds of being overweight or obese at 0–8 years (pooled odds ratio from 10 studies = 1.32, 95% CI = 1.15–1.51) [8]. Another review also determined CS children to be at higher risk of being obese at 2–18 years (pooled risk ratio from 19 studies = 1.34, 95% CI = 1.18–1.51) [9].

Nine other studies provide evidence to suggest delivering by CS increases the risk of child adiposity [19–27]. However, three studies have found no evidence of differences in child BMI between CS and vaginal deliveries [28–30].

A further search found one study to address the effects of ethnicity on the association of mode of delivery and child adiposity. The study found differing race-specific effects of CS with body size at 2 years between African American and non-African American mothers [26]. In children of African American mothers, CS was associated with a significantly higher odds of obesity, whereas no association was found in children of non-African American mothers [26]. It was suggested that ethnic differences in the developing gut microbiome or epigenetic structure, could be the cause of the effect modification [26].

In this paper, studies were cited if they reported effect estimates for the association between the mode of delivery (CS compared with vaginal delivery) and overweight or obesity in children. The age range defining childhood was 2–18 years.

### Rationale for this study

There is limited published research on the direct association of mode of delivery and child BMI, at the age of children starting school (4–5 years). This association has not been investigated in UK South Asian mothers compared to WB mothers.

The aim of this study was to determine if there is any association between mode of delivery (CS and vaginal delivery) and BMI at 4–5 years of age, in the Born in Bradford (BiB) cohort, and if this differs between ethnicities (WB and Pakistani).

## Methods

### Study design

Born in Bradford (BiB) is a longitudinal multi-ethnic birth cohort study. BiB aims to investigate parent and child wellbeing by examining physiological, environmental and genetic factors in the City of Bradford [31]. Bradford is situated in the north of England; it is ethnically diverse and has high levels of socio-economic deprivation. BiB recruitment occurred from September 2007 to December 2010. Women who attended the Bradford Royal Infirmary at 26–28 weeks gestation for a routine glucose-tolerance test, which is offered to all women booked to give birth in Bradford, were invited to join the study and 87% of those approached agreed to participate. It is at this point that women were weighed, and their height measured. Weight at first antenatal clinic assessment (median 12 weeks’ gestation) was extracted from the antenatal records and this weight, together with height measured at recruitment, was used to calculate the woman’s early pregnancy BMI (kg/m^2^).

The BiB population is broadly representative of the maternal population in Bradford [31]. Twelve thousand four hundred fifty-three pregnant women gave consent to be involved with the study. One woman could contribute more than a single pregnancy, resulting in a total of 13,776 pregnancies being reported, which gave rise to 13,858 children. A baseline questionnaire was conducted at recruitment. The mother self-reported most variables, including ethnicity, socioeconomic indicators, alcohol-related and smoking habits. In this analysis, the mother’s self-reported ethnicity was used to define their child’s ethnicity. School nurse teams took anthropometric measurements of children in reception class (aged 4–5) as part of the National Child Measurement Programme (NCMP). Where anthropometric measurements were missing from NCMP data, it was possible to obtain some of these measurements from Primary Care and Child Health Records at this age. Of the non-missing anthropometric data, 82.2% originated from NCMP, 10.2% from Primary Care, 7.6% from Child Health Records. A subgroup of mothers was followed up for data on breastfeeding, at 6, 12, 18, 24 and 36 months. This subgroup was part of BiB1000, a nested cohort of the BiB prospective birth cohort [32].

### Study population

From the total 13,858 children enrolled in BiB, 7448 were excluded due to not meeting the inclusion criteria of having data on mode of delivery, BMI at 4–5 years, singleton birth and being WB or Pakistani. As approximately a third of the children enrolled in BiB are missing outcome data (BMI at 4–5 years), a characteristics table comparing the final study population (*n* = 6410) and those with missing BMI at 4–5 years (*n* = 4154) is presented in the supplementary material. There is no suggestion of selection bias at this stage.

**Fig. 1:**
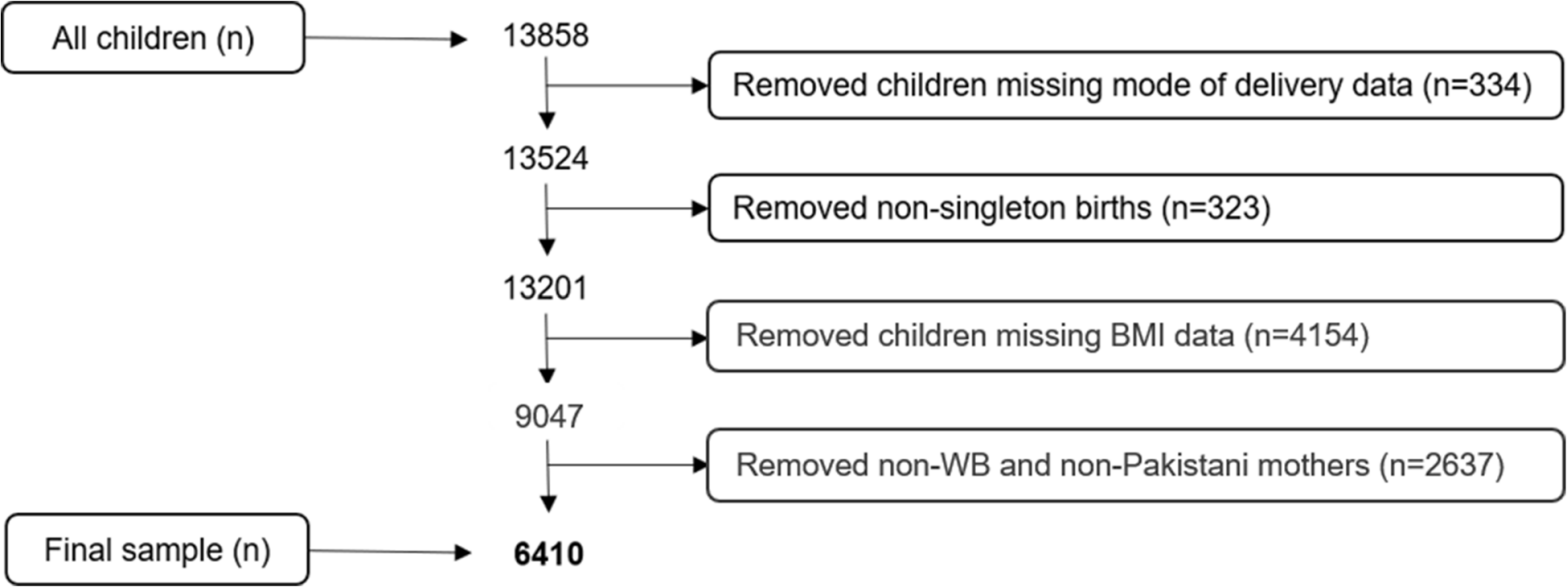
A flowchart describing the selection of the final study population. Abbreviation: WB, White British

Mothers self-reporting an ethnicity other than WB or Pakistani were excluded as they were a very heterogeneous ethnic group (429 = Indian, 288 = White Other, 253 = Bangladeshi, 226 = Black, 108 = Mixed-White and Black, 61 = Mixed-White and South Asian, 309 = other). This left a sample size of 6410 children (Fig. 1). The original BiB cohort had a similar distribution of child BMI and mode of delivery to the study population.

### Exposure (mode of delivery)

Exposed participants were children who were delivered by elective or emergency CS. Unexposed participants were children who were delivered vaginally; including normal, forceps and ventouse extraction deliveries. Mode of delivery was recorded by a midwife or paediatrician within the first 6 h of life. Paper forms with handwritten notes were entered into the routine eClipse electronic maternity record as neonatal data.

### Outcome (child BMI)

BMI values of children aged 4–5 years, recorded as part of the NCMP by school nurse teams [33] or obtained from Primary Care or Child Health Records, were transformed to a standardised measure (z-scores). The z-scores were calculated using the LMS method. This is prepared via an Excel spreadsheet, which can be obtained online for free [34]. The LMS growth application includes access to a 1990 UK (UK90) reference population. Using this reference, each individual is assigned a z-score which adjusts for age, sex and the BMI distribution for skewness [35]. The UK90 reference group is recommended for population monitoring and clinical assessment in children aged 4 years and over [36]. It serves as an anchor for comparison; it is used by the NCMP and has been used for other BiB studies [37]. Children with BMI z-scores above the 85th percentile were classified as overweight, and those above the 95th percentile as obese [38].

### Sample size calculations

Sample size calculations were conducted in OpenEpi [39]. This study had a power of 99%, determined from a post-hoc power calculation using the parameters from this study (vaginal to CS ratio of 3.71 and prevalence of childhood overweight or obesity in vaginal births at 14.8%), and the odds ratio from a previous study (odds ratio of 2.10 (95% CI 1.36–3.23) of obesity in children aged 7 years, by CS compared to vaginal birth) [10].

### Statistical analysis

All analyses were conducted in Stata/IC 15.1. Figures were produced using RStudio version 1.3.1056. Variables that had good evidence (chi-squared tests, ANOVA, and judging correlation to have approximately *p* < 0.05) to suggest they had an association with both mode of delivery and z-scores, as well as not being on the causal pathway, met the criteria to be potential confounders. The following variables were considered for assessment of being potential confounders: maternal age; maternal BMI; maternal education; maternal job status; maternal house tenure; maternal benefits received; maternal drinking of alcohol during pregnancy or 3 months before; maternal smoking during pregnancy; parity; maternal gestational diabetes; child gender; child birthweight; gestational period.

### Multivariable analysis

The forward selection approach was used to create regression models. Potential confounders were added individually according to their effect size. The covariate was retained in the model if there was an appreciable (10%) difference in effect size of mode of delivery on z-score.

The final multivariable linear regression model assessed the association between mode of delivery and BMI z-score. Preliminary analysis confirmed the assumptions of the regression were met; z-scores were normally distributed and lacked collinearity. BMI z-scores were also categorised and logistic regression models performed to obtain odd ratios for being overweight and obese in children delivered by CS. All the study population (*n* = 6410) contributed to the unadjusted regression modeling. Complete case analysis was used for the adjusted regression modeling (*n* = 6115).

### Effect modification

Potential effect modification was judged by stratifying the final model by ethnicity to observe the separate association of mode of delivery on z-score in WB and Pakistani children. A formal test for effect modification was also conducted; a likelihood ratio test compared the final model with a model which also included an interaction term between mode of delivery and ethnicity.

### Missing data

The number and proportion of patients missing data on descriptive variables was described. Complete case analysis was used for the multivariate analysis. No imputation was performed.

Approximately 85% of the study population had missing data on breastfeeding (*n* = 5439 missing). Due to the large proportion of missingness, breastfeeding was not assessed in this study. Maternal parity (*n* = 273 missing) and maternal BMI (*n* = 275 missing) were missing for about 4% of the study population.

## Results

### Descriptive results

**Fig. 2:**
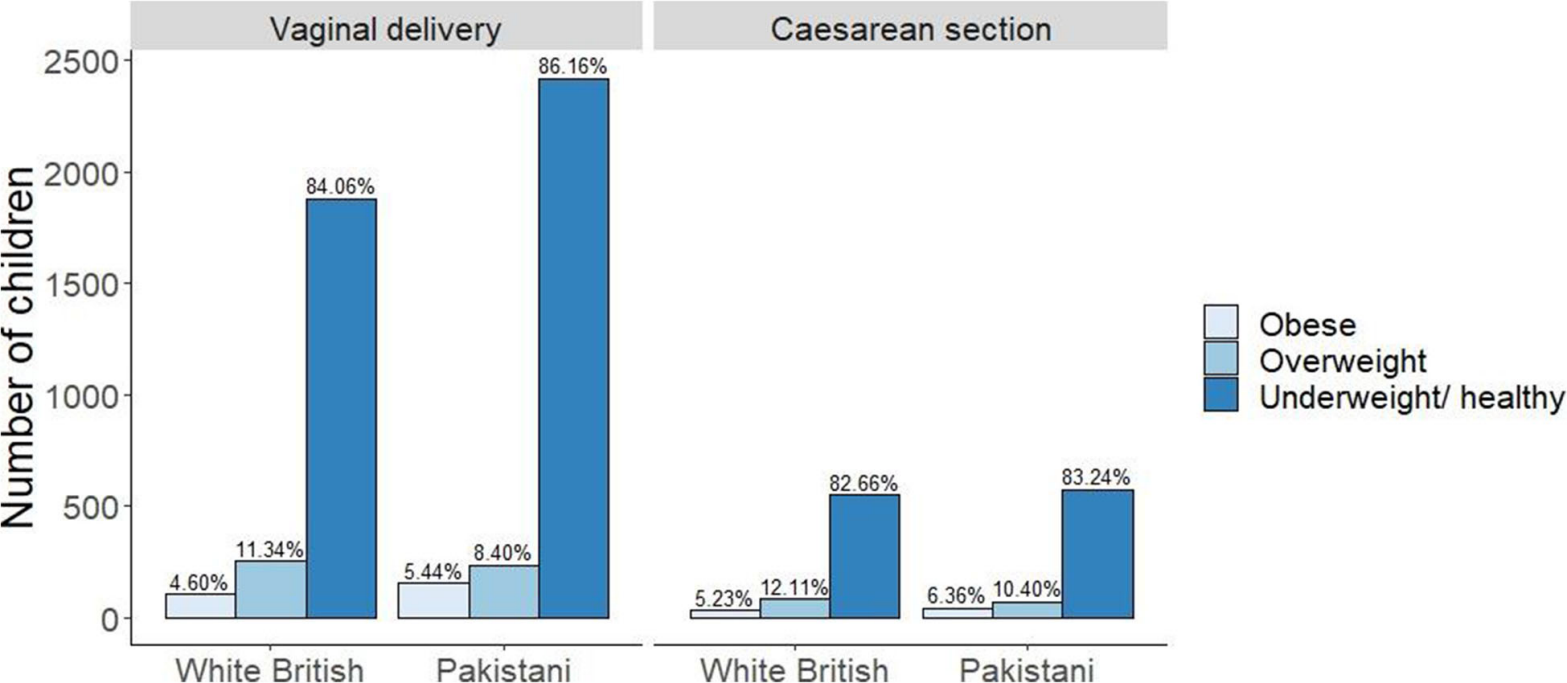
A bar chart showing the number of children in the study population, stratified by mode of delivery, ethnicity and categorised BMI

Tables 1 and 2 summarise the baseline characteristics of the study population stratified by mode of delivery and ethnicity, also visualised in Fig. 2. In this study, 21.3% (*n* = 1361) of babies were delivered by CS. There were more Pakistani mothers (54.6%, *n* = 3502) than WB mothers (45.4%, *n* = 2908). Amongst Pakistani mothers, 19.8% had CS, whereas the CS prevalence among WB mothers was higher at 23.0%. Most children were underweight/ healthy weight (84.7%), and fewer were overweight (10.0%) or obese (5.2%) (Table 2). The mean BMI z-score was slightly higher in CS deliveries (0.32) than vaginal deliveries (0.22) at age 4–5 years. Furthermore, the mean BMI z-score was higher in WB children (0.43) than Pakistani children (0.08).

**Table 1:** Baseline characteristics, of the study population, stratified by mode of delivery

		Vaginal (*n* = 5049)		Caesarean (*n* = 1361)		*P* value
		n	%	n	%	
Child BMI categorised (*n* = 6410)	Underweight/Healthy weight	4303	85.22	1129	82.95	0.039
	Overweight	490	9.70	153	11.24	
	Obese	256	5.07	79	5.80	
Child BMI z-score (*n* = 6410)	Mean	0.22		0.32		0.003
	SD	1.11		1.15		
Ethnicity (*n* = 6410)	White British	2239	44.35	669	49.16	0.002
	Pakistani	2810	55.65	692	50.84	
Maternal age (years) (*n* = 6410)	Mean	27.12		28.77		< 0.0001
	SD	5.53		5.73		
Maternal BMI at early pregnancy categorised (*n* = 6135)	Underweight/Healthy weight	2502	51.83	517	39.53	< 0.0001
	Overweight	1414	29.29	395	30.20	
	Obese	911	18.87	396	30.28	
Maternal BMI at early pregnancy (kg/m^2^) (*n* = 6135)	Mean	25.67		27.67		< 0.0001
	SD	5.46		6.26		
Maternal education (*n* = 6395)	< 5 GCSE equivalent	1230	24.41	272	20.04	< 0.001
	5 GCSE equivalent	1718	34.10	437	32.20	
	A-level equivalent	695	13.80	181	13.34	
	Higher than A-level	1058	21.00	362	26.68	
	Foreign unknown/other	337	6.69	105	7.74	
Maternal job status (*n* = 6400)	Currently employed	2029	40.24	678	49.93	< 0.001
	Previously employed	1517	30.09	368	27.10	
	Never employed	1496	29.67	312	22.97	
Maternal house tenure (*n* = 6396)	Owns outright	807	16.02	207	15.25	0.055
	Mortgage	2518	49.97	718	52.91	
	Private landlord	792	15.72	172	12.68	
	Social housing	522	10.36	145	10.69	
	Rent free/other	400	7.94	115	8.47	
Maternal benefits received (*n* = 6385)	Yes	2237	44.51	496	36.50	< 0.001
	No	2789	55.49	863	63.50	
Maternal drinking of alcohol during pregnancy or 3 months before (*n* = 6393)	Yes	1570	31.17	495	36.42	0.001
	No	3465	68.79	863	63.50	
Maternal smoking during pregnancy (*n* = 6396)	Yes	856	16.99	210	15.45	0.176
	No	4181	83.01	1149	84.55	
		211	27.47	59	29.06	
Parity (*n* = 6137)	Primiparous	1802	37.22	586	45.22	< 0.001
	Multiparous	3039	62.78	710	54.78	
Maternal gestational diabetes (*n* = 6404)	Yes	342	6.78	147	10.83	< 0.001
	No	4705	93.22	1210	89.17	
Child gender (*n* = 6410)	Male	2548	50.47	718	52.76	0.134
	Female	2501	49.53	643	47.24	
Child birthweight (g) (*n* = 6410)	Mean	3244.10		3215.84		0.089
	SD	503.61		675.73		
Gestational period (days) (*n* = 6410)	Mean	277.67		273.90		< 0.0001
	SD	11.09		15.34		
Gestational period (*n* = 6410)	Preterm (< 37 weeks)	208	4.12	117	8.60	< 0.0001
	Term (≥37 weeks)	4841	95.88	1244	91.40	

*P* values to provide the level of statistical evidence on the difference between mode of delivery; obtained from chi-squared tests or ANOVA, where appropriate

*Abbreviations*: *n* sample size, *BMI* body mass index, *SD* standard deviation

**Table 2:** Baseline characteristics, of the study population, stratified by ethnicity

		All		White British (*n* = 2908)		Pakistani (*n* = 3502)		*P* value
		n	%	n	%	n	%	
Child BMI categorised (*n* = 6410)	Underweight/Healthy weight	5432	84.74	2435	83.73	2997	85.58	0.041
	Overweight	643	10.03	335	11.52	308	8.79	
	Obese	335	5.23	138	4.75	197	5.63	
Child BMI z-score (*n* = 6410)	Mean	0.24		0.43		0.08		< 0.0001
	SD	1.12		0.97		1.21		
Maternal age (years) (*n* = 6410)	Mean	27.47		27.03		27.84		< 0.0001
	SD	5.61		6.09		5.15		
Maternal BMI at early pregnancy categorised (*n* = 6135)	Underweight/Healthy weight	3019	49.21	1269	45.78	1750	52.04	< 0.0001
	Overweight	1809	29.49	810	29.22	999	29.71	
	Obese	1307	21.30	693	25.00	614	18.26	
Maternal BMI at early pregnancy (kg/m^2^) (*n* = 6135)	Mean	26.09		26.80		25.50		< 0.0001
	SD	5.69		5.97		5.39		
Maternal education (*n* = 6395)	< 5 GCSE equivalent	1502	23.49	577	19.87	925	26.50	< 0.001
	5 GCSE equivalent	2155	33.70	1028	35.40	1127	32.28	
	A-level equivalent	876	13.70	458	15.77	418	11.97	
	Higher than A-level	1420	22.20	560	19.28	860	24.63	
	Foreign unknown/other	442	6.91	281	9.68	161	4.61	
Maternal job status (*n* = 6400)	Currently employed	2707	42.30	1924	66.21	783	22.41	< 0.001
	Previously employed	1885	29.45	775	26.67	1110	31.77	
	Never employed	1808	28.25	207	7.12	1601	45.82	
Maternal house tenure (*n* = 6396)	Owns outright	1014	15.85	119	4.10	895	25.63	< 0.001
	Mortgage	3236	50.59	1519	52.31	1717	49.17	
	Private landlord	964	15.07	654	22.52	310	8.88	
	Social housing	667	10.43	451	15.53	216	6.19	
	Rent free/other	515	8.05	161	5.54	354	10.14	
Maternal benefits received (*n* = 6385)	Yes	2733	42.80	1063	36.73	1670	47.84	< 0.001
	No	3652	57.20	1831	63.27	1821	52.16	
Maternal drinking of alcohol during pregnancy or 3 months before (*n* = 6393)	Yes	2065	32.29	2055	70.72	10	0.29	< 0.001
	No	4328	67.67	848	29.18	3480	99.71	
Maternal smoking during pregnancy (*n* = 6396)	Yes	1066	16.67	961	33.08	105	3.01	< 0.001
	No	5330	83.33	1944	66.92	3386	96.99	
Parity (*n* = 6137)	Primiparous	2388	38.91	1351	48.11	1037	31.15	< 0.001
	Multiparous	3749	61.09	1457	51.89	2292	68.85	
Maternal gestational diabetes (*n* = 6404)	Yes	489	7.64	141	4.85	348	9.95	< 0.001
	No	5915	92.36	2764	95.15	3151	90.05	
Child gender (*n* = 6410)	Male	3266	50.95	1487	51.13	1779	50.80	0.789
	Female	3144	49.05	1421	48.87	1723	49.20	
Child birthweight (g) (*n* = 6410)	Mean	3238.10		3357.60		3138.86		< 0.0001
	SD	544.78		550.53		519.56		
Gestational period (days) (*n* = 6410)	Mean	276.86		277.63		276.22		< 0.0001
	SD	12.22		12.51		11.94		
Gestational period (*n* = 6410)	Preterm (< 37 weeks)	325	5.07	157	5.40	168	4.80	
	Term (≥37 weeks)	6085	94.93	2751	94.60	3334	95.20	0.300

*P* values to provide the level of statistical evidence on the difference between White British and Pakistani ethnic groups; obtained from chi-squared tests or ANOVA, where appropriate

*Abbreviations*: *n* sample size, *BMI* body mass index, *SD* standard deviation

### Mother’s booking BMI

Almost half the mothers in this study population were underweight or healthy weight (49.2%). Obesity was more prevalent in women who gave birth by CS (30.3%), compared to mothers giving birth vaginally (18.9%). WB mothers had a slightly higher BMI (+ 1.30 kg/m^2^) than Pakistani women. Leading to a higher prevalence of obesity in WB mothers (25.0%) than Pakistani women (18.2%).

### Family sociodemographic factors

Mothers who had CS often had achieved a higher level of education than mothers with vaginal deliveries (26.7% of CS mothers, 21.0% of vaginal mothers). More Pakistani women achieved higher than A-level qualifications compared to WB women (24.6 vs 19.3%).

Most mothers were not currently employed (67.7%). Current employment was more common in women who had CS births, compared to those with vaginal births (49.9 and 40.2%, respectively) and also more common in WB women (66.2%), compared to Pakistani women (22.4%). Additionally, a higher proportion of mothers having vaginal deliveries (44.5%) received benefits than those having CS deliveries (36.5%). Receiving benefits was more common in Pakistani mothers (47.8%) than WB mothers (36.7%).

### Gestational factors

The mean age of mothers who had a CS was 28.8 years old, which was 1.7 years older than those who gave birth vaginally. Also, mothers having vaginal deliveries were more likely to be multiparous (62.8%) compared to mothers having CS deliveries (54.8%). The difference in mean gestation period between CS and vaginal deliveries was minimal (4 days difference). A larger proportion of preterm births was experienced by mothers who had a CS (8.6%), compared to mothers who gave birth vaginally (4.1%).

Only 10 out of 3490 Pakistani mothers (0.3%) drank alcohol during pregnancy or 3 months before, whereas 70.7% of WB mothers reported alcohol consumption. Further to this, mothers having CS were marginally more likely to have drunk alcohol during pregnancy or 3 months before (36.4% of CS mothers and 31.2% of vaginal mothers). More WB women reportedly smoked during pregnancy than Pakistani women (33.1% of WB women, 3.0% of Pakistani women).

Gestational diabetes was more prevalent in mothers having CS (10.8%) compared to those having vaginal deliveries (6.8%). Additionally, prevalence was higher in Pakistani mothers than WB mothers (10.0 and 4.9% respectively).

### Child factors

Children born by CS had a mean birthweight 28.3 g lower than vaginal births. Irrespective of mode of delivery, children with WB mothers had a higher mean birthweight than those with Pakistani mothers (3357.6 g and 3138.9 g, respectively).

### Multivariable analysis

**Fig. 3:**
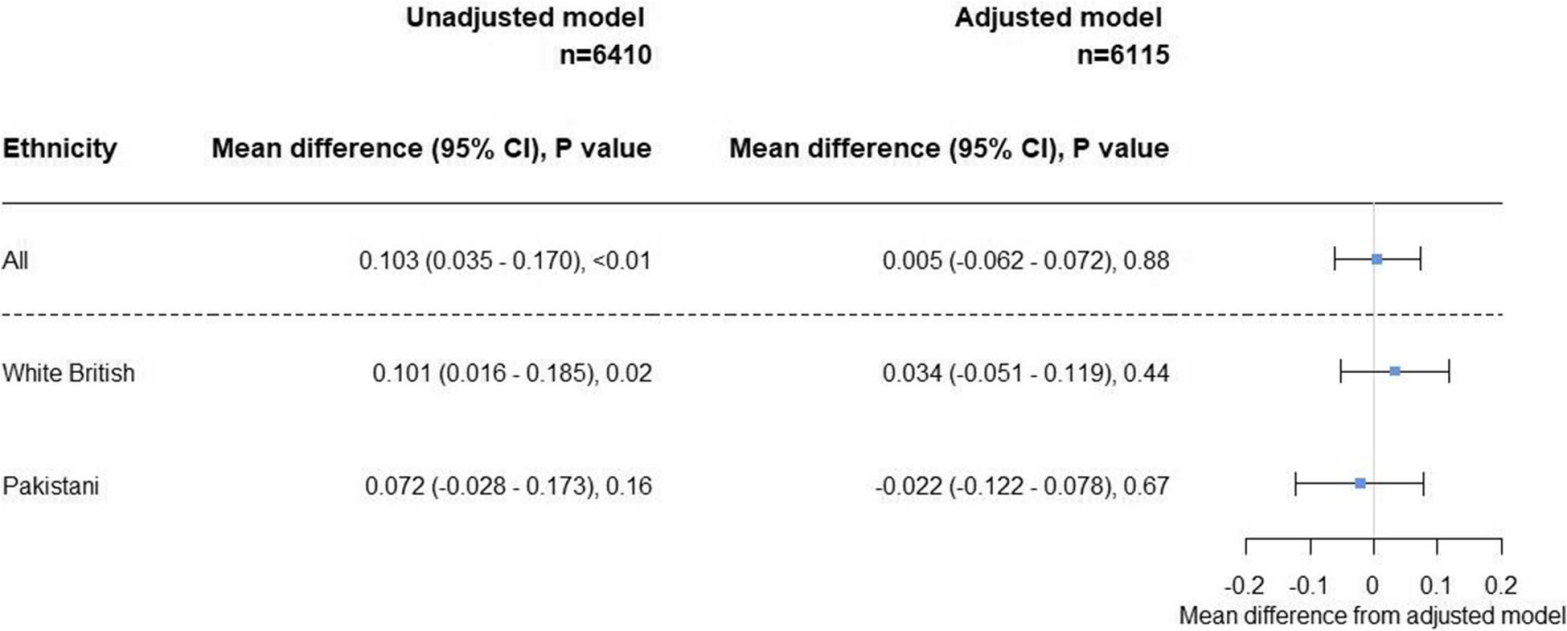
Mean differences in BMI z-score depending on mode of delivery, at 4–5 years old, from White British and Pakistani ethnic groups. The reference group had vaginal deliveries. Values obtained are β coefficients from unadjusted and adjusted linear regression models. *P* value acquired from t-tests. The adjusted model controls for: ethnicity, maternal BMI (continuous), maternal job status and drinking alcohol during or 3 months before pregnancy

The unadjusted linear regression calculated the predicted difference in z-score between mode of delivery, the z-score being higher with CS (*n* = 6410, difference = 0.103; 95% CI = 0.035–0.170) (Fig. 3). The adjusted model calculated the predicted mean difference in z-score between mode of delivery, controlling for all factors which met the confounding criteria (ethnicity, maternal BMI (continuous), maternal job status and maternal drinking of alcohol during pregnancy or 3 months before), there was no difference in child BMI z-score (*n* = 6115, difference = 0.005; 95% CI = −0.062–0.072) (Fig. 3).

The proportion of variance in BMI z-scores explained by the mode of delivery was 7.49% for the adjusted model (adjusted *R*^2^ value). The F ratio (71.76) shows how much variability the model can explain relative to how much it cannot explain. The standard error (0.034) did not differ between unadjusted and adjusted models, suggesting an absence of collinearity.

**Fig. 4:**
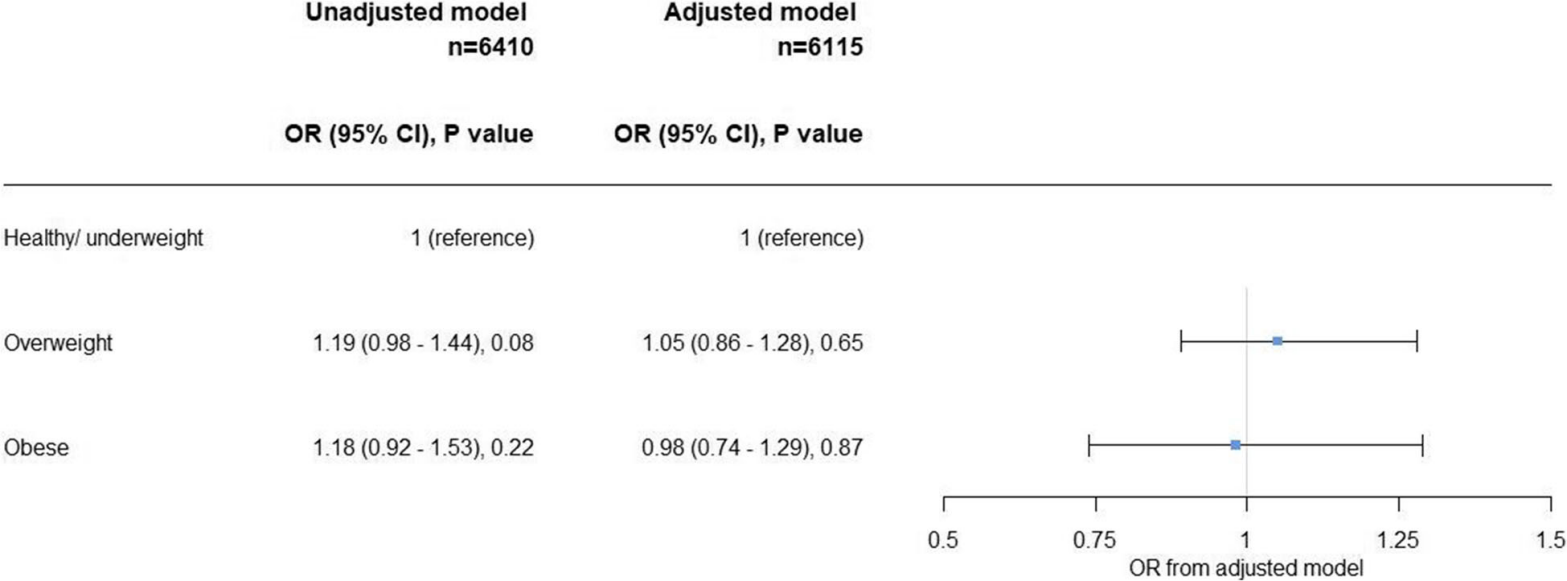
Results obtained from logistic regression models, for the association between categorised BMI z-score in children delivered by caesarean section, at 4–5 years old, from White British and Pakistani ethnic groups. Adjusted for: ethnicity, maternal BMI (continuous), maternal job status and drinking alcohol during or 3 months before pregnancy. Abbreviations: OR, odds ratio; CI, confidence intervals

The adjusted logistic regression models with the outcome of overweight and obesity obtained odds ratios and confidence intervals of no strong statistical support for a difference in odds between mode of delivery (Fig. 4).

### Effect modification

Stratified analysis suggests the adjusted association of mode of delivery on BMI z-score is similar, irrespective of ethnicity (Fig. 3). Additionally, there was no evidence of effect modification from the likelihood ratio test (*p* = 0.97). When a test for effect modification was performed on the categorised z-scores, similar results were obtained. There was weak evidence of effect modification by ethnicity on the association between mode of delivery and overweight (*p* = 0.14), and no evidence for effect modification by ethnicity on the association between mode of delivery and obesity (*p* = 0.79).

## Discussion

In this cohort study, it was found that undergoing a CS was not associated with an increased risk of overweight and obesity in children, and there was no difference between ethnic groups.

Mothers who had undergone a CS were generally of higher socio-economic status than those who had vaginal deliveries; CS women were more educated, more likely to be currently employed, more likely to have a stable housing situation and less likely to be receiving benefits. Also, mothers who experienced CS had baseline characteristics to suggest they had poorer health than mothers giving birth vaginally; CS mothers had a higher mean BMI, were more likely to drink alcohol during pregnancy or 3 months before and more prevalent gestational diabetes. The distribution of alcohol drinking varies vastly between ethnicities, this is most likely due to religious beliefs [31]. This explains the very low prevalence of alcohol drinking and avoidance of smoking amongst Pakistani women.

The linear regression for the adjusted model offers no evidence for a difference in BMI z-score between children born via CS and vaginal deliveries. The low adjusted *R*^2^ value suggests there are other variables which have an influence on the primary association. The adjusted logistic regression models also suggest no evidence for children delivered by CS having different odds of being overweight or obese, compared to children of vaginal deliveries.

The stratified analysis and formal test for effect modification both indicate there is no evidence that the association between mode of delivery and children’s BMI z-scores varied by ethnicity.

As discussed in the introduction, previous studies have varied interpretations. Two leading systematic reviews suggest there is evidence that CS increases child BMI [8, 9]. However, there were several studies which found no statistical association between mode of delivery and child BMI. The findings from this paper are compatible with the latter studies mentioned.

Two out of three studies conducted in the UK concluded there was no ‘statistical significant’ difference in risk of childhood overweight or obesity between modes of delivery, at 3 years old [40] and 5 years old [28].

Furthermore, maternal BMI explained most of the observed association in this study and was hence the main confounding factor. All previous studies cited here, looking at the association between mode of delivery and BMI, also adjusted for this factor.

However, this study also differs with previous research. The other UK study found that CS increased the odds of being overweight or obese, at 7 years old [19]. This was a study which used data from the Avon Longitudinal Study of Parents and Children (ALSPAC); participants were recruited from the Avon area if they were born in 1991–1992.

Several confounders (child gender, gestational factors and child feeding patterns) were adjusted for in the ALSPAC study but did not meet the confounding criteria (or the data were unavailable) to be adjusted for in this study. There were also inconsistencies with other factors adjusted for in this study compared to previous studies, such as not adjusting for antibiotics during pregnancy [20]. Different factors could have met the confounding criteria in previous studies due to their population type, for example, by having a different BMI distribution as the children were leaner.

Adjusting for ethnicity was not seen in previous research in the UK. Due to the large proportion of Pakistani women in this BiB study, there was sufficient power to investigate differences between WB and Pakistani ethnic groups, whereas this would not be possible in studies like ALSPAC. As previous studies did not adjust for ethnicity, other variables could have acted as confounders. Overall, the differences in study design, study population and confounding adjustments could explain the inconsistent conclusions reached.

The large sample size used in this study allowed sufficient power to identify any meaningful differences in association between BMI z-scores of two different modes of delivery. Additionally, consistent statistical methodology with previous studies was used and there was minimal recruitment bias due to the BiB study having a high recruitment rate of 87%.

As CS and vaginal deliveries are very different procedures, in theory, there was no opportunity for this to be incorrectly recorded. Hence no information bias, in the form of non-differential misclassification, should have occurred. Furthermore, observer bias would not arise when recording the child’s BMI, as nurses taking anthropometric measurements at ages 4–5 were blinded to information regarding the child’s mode of delivery.

There is evidence to suggest BMI measurements systematically underestimate childhood adiposity [41]. This has also been specifically investigated in South Asians with evidence to suggest that BMI additionally appears to underestimate adiposity in this ethnic group. Despite South Asians being generally smaller and lighter, they seem to have greater relative fatness compared to white European populations [42].

Most of the data on covariates were collected in the baseline questionnaire, completed by the mother. As data were self-reported, information bias in the form of differential misclassification could have occurred which would tend the results to overestimate or underestimate the true association. An example would be smoking as this is a likely factor to be underreported. Underreporting could underestimate the association between mode of delivery and smoking, which would have led to it not being adjusted for in the final analysis.

There may be residual confounding which is obscuring the true effect of mode of delivery on child BMI. The low adjusted *R*^2^ value implies other factors could have an influence on the association, therefore suggesting factors which were not included in the analysis explained some of the association. These could be factors such as amount of exercise feeding pattern of the child or breastfeeding. It would have been desirable to have considered breastfeeding as a potential confounder but there was insufficient data to assess this.

The study was limited by approximately 85% of the study population having missing data on breastfeeding due to the data being collected in a subgroup of women who participated in the BiB1000, as described in the study design section. Parity and maternal BMI was also missing for 4% of the final study population. There was no evidence of any statistically significant difference in the distribution of mode of delivery or child BMI z-score, when comparing: those with data on parity vs those missing parity; and those with data on maternal BMI vs those missing maternal BMI. Therefore, there was no evidence of selection bias based on the distribution of missing data in parity and maternal BMI.

Additionally, these results will be generalisable to other populations with similar demography to Bradford. The CS rate in Bradford is not markedly different to the national rate, and obesity at 4–5 years is very slightly above the national average (10.8% in Bradford, 9.9% in England, in 2019–2020) [12]. The results obtained from the Pakistani population may not be generalisable to other South Asian groups.

## Conclusion

Overall CS was not associated with an increased risk of overweight or obesity in children aged 4–5 years in Bradford. Neither was there a difference in association seen between White British or Pakistani children. To our knowledge, this is the first study to assess this association, between these ethnicities, at this age.

As CS deliveries are becoming more common globally and health concerns have been raised, the results from this study, combined with similar studies, should be informative to prospective parents and healthcare advisors.

Data collection within the BiB cohort should be continued to provide more reliable estimates of adiposity and to allow investigation at older ages. This will enable examination of whether any association exists at subsequent ages between mode of delivery and later life adiposity.

As there is some uncertainty around how well BMI represents child adiposity, the use of body fat centile curves should be explored instead. To do this, data on fat mass analysed using a DXA scanner would be needed. A DXA scanner is an extremely accurate method for analysing body composition, and could be used as a gold standard in the population. The sensitivity and specificity of BMI against the DXA scanner in the population can be obtained and incorporated in the interpretation.

Further research could also investigate if the method of fetus extraction acts as an effect modifier on the association between mode of delivery and BMI. Vaginal deliveries can occur by: natural vaginal birth, forceps assistance or by ventouse techniques. CS can occur by: emergency, elective or semi-elective delivery. These studies would need to have a large sample size to power the subgroup analyses.

Additionally, if the mechanism for any potential association is related to the developing gut microbiota, then more studies could focus on the differences between gut microbiota stratified by mode of delivery and by ethnicity. This is likely to involve a genetic approach when looking at the differences between the WB and Pakistani population [17].

## Supplementary Information

**Additional file 1.** Characteristics of the missing outcome observations compared with the final sample population.

## Data Availability

Data from the BiB study is available to researchers following approval from the Executive Committee (https://borninbradford.nhs.uk/research/how-to-access-data/).

## Abbreviations

ALSPAC: Avon Longitudinal Study of Parents and Children
BiB: Born in Bradford
BMI: Body mass index
CI: Confidence intervals
CS: Caesarean section
DXA: Dual-energy X-ray absorptiometry
NCMP: National Child Measurement Programme
OR: Odds ratio
WB: White British
