# Supplementary material for: Association between mode of delivery and body mass index at 4-5 years in White British and Pakistani children: the Born in Bradford birth cohort
Source: BMC Public Health. 2021 May 26;21:987. doi: 10.1186/s12889-021-11009-y (PMC8152119; doi:10.1186/s12889-021-11009-y)
Supplement: Supplementary file 1 — Additional file 1. Characteristics of the missing outcome observations compared with the final sample population. [file 12889_2021_11009_MOESM1_ESM.docx]

**Supplementary Material- Characteristics of the missing outcome observations compared with the final sample population.**

|  |  | Final study population  (n= 6410) | | Population missing child BMI  (n= 4154) | |
| --- | --- | --- | --- | --- | --- |
|  |  | **n** | **%** | **n** | **%** |
| Child BMI categorised | Underweight/  Healthy weight | 5432 | 84.74 | Missing | |
|  | Overweight | 643 | 10.03 |  |  |
|  | Obese | 335 | 5.23 |  |  |
| Child BMI z-score | N | 6410 | | Missing | |
|  | Mean (SD) | 0.24 (1.12) | |  |  |
| Ethnicity | White British | 2908 | 45.37 | 1400 | 49.37 |
|  | Pakistani | 3502 | 54.63 | 1436 | 50.63 |
| Maternal age (years) | N | 6410 | | 4154 | |
|  | Mean (SD) | 27.47 (5.61) | | 27.54 (5.65) | |
| Maternal BMI at early pregnancy categorised | Underweight/  Healthy weight | 3019 | 49.21 | 1597 | 48.93 |
|  | Overweight | 1809 | 29.49 | 951 | 29.14 |
|  | Obese | 1307 | 21.30 | 716 | 21.94 |
| Maternal BMI at early pregnancy (kg/m^2^) | N | 6135 | | 3264 | |
|  | Mean (SD) | 26.09 (5.69) | | 26.16 (5.78) | |
| Maternal education | <5 GCSE equivalent | 1502 | 23.49 | 708 | 20.30 |
|  | 5 GCSE equivalent | 2155 | 33.70 | 985 | 28.24 |
|  | A-level equivalent | 876 | 13.70 | 547 | 15.68 |
|  | Higher than A-level | 1420 | 22.20 | 991 | 28.41 |
|  | Foreign unknown/other | 442 | 6.91 | 257 | 7.37 |
| Maternal job status | Currently employed | 2707 | 42.30 | 1537 | 44.08 |
|  | Previously employed | 1885 | 29.45 | 951 | 27.27 |
|  | Never employed | 1808 | 28.25 | 999 | 28.65 |
| Maternal house tenure | Owns outright | 1014 | 15.85 | 389 | 11.17 |
|  | Mortgage | 3236 | 50.59 | 1333 | 38.26 |
|  | Private landlord | 964 | 15.07 | 876 | 25.14 |
|  | Social housing | 667 | 10.43 | 390 | 11.19 |
|  | Rent free/other | 515 | 8.05 | 496 | 14.24 |
| Maternal benefits received | Yes | 2733 | 42.80 | 1401 | 40.21 |
|  | No | 3652 | 57.20 | 2083 | 59.79 |
| Maternal drinking of alcohol during pregnancy or 3 months before | Yes | 2065 | 32.30 | 1022 | 29.33 |
|  | No | 4328 | 67.70 | 2462 | 70.67 |
| Maternal smoking during pregnancy | Yes | 1066 | 16.67 | 624 | 17.88 |
|  | No | 5330 | 83.33 | 2865 | 82.12 |
| Parity | Primiparous | 2388 | 38.91 | 1709 | 42.38 |
|  | Multiparous | 3749 | 61.09 | 2324 | 57.62 |
| Maternal gestational diabetes | Yes | 489 | 7.64 | 356 | 8.65 |
|  | No | 5915 | 92.36 | 3760 | 91.35 |
| Child gender | Male | 3266 | 50.95 | 2143 | 51.59 |
|  | Female | 3144 | 49.05 | 2011 | 48.41 |
| Child birthweight (g) | N | 6410 | | 4153 | |
|  | Mean (SD) | 3238.10 (544.78) | | 3236.07 (584.36) | |
| Gestational period (days) | N | 6410 | | 4154 | |
|  | Mean (SD) | 276.86 (12.22) | | 276.32 (14.06) | |
| Gestational period | Preterm (<37 weeks) | 325 | 5.07 | 274 | 6.60 |
|  | Term (≥37 weeks) | 6085 | 94.93 | 3880 | 93.40 |
